# Fumaric Acid Esters Stimulate Astrocytic VEGF Expression through HIF-1α and Nrf2

**DOI:** 10.1371/journal.pone.0076670

**Published:** 2013-10-03

**Authors:** Diana Wiesner, Irma Merdian, Jan Lewerenz, Albert C. Ludolph, Luc Dupuis, Anke Witting

**Affiliations:** 1 Department of Neurology, University of Ulm, Ulm, Germany; 2 U1118 Mécanismes centraux et périphériques de la neurodégénérescence, Inserm, Strasbourg, France; 3 UMRS1118, Université de Strasbourg, Fédération de médecine translationnelle, Strasbourg, France; Penn State Hershey Medical Center, United States of America

## Abstract

Fumaric acid esters (FAE) are oral analogs of fumarate that have recently been shown to decrease relapse rate and disease progression in multiple sclerosis (MS), prompting to investigate their protective potential in other neurological diseases such as amyotrophic lateral sclerosis (ALS). Despite efficacy in MS, mechanisms of action of FAEs are still largely unknown. FAEs are known to activate the transcription factor Nrf2 and downstream anti-oxidant responses through the succination of Nrf2 inhibitor KEAP1. However, fumarate is also a known inhibitor of prolyl-hydroxylases domain enzymes (PhD), and PhD inhibition might lead to stabilization of the HIF-1α transcription factor under normoxic conditions and subsequent activation of a pseudo hypoxic response. Whether Nrf2 activation is associated with HIF-1α stabilization in response to FAEs in cell types relevant to MS or ALS remains unknown. Here, we show that FAEs elicit HIF-1α accumulation, and VEGF release as its expected consequence, in astrocytes but not in other cell types of the central nervous system. Reporter assays demonstrated that increased astrocytic VEGF release in response to FAEs was dependent upon both HIF-1α and Nrf2 activation. Last, astrocytes of transgenic mice expressing SOD1(G93A), an animal model of ALS, displayed reduced VEGF release in response to FAEs. These studies show that FAEs elicit different signaling pathways in cell types from the central nervous system, in particular a pseudo-hypoxic response in astrocytes. Disease relevant mutations might affect this response.

## Introduction

Fumaric acid esters (FAE) are oral analogs of fumarate and have been used in the treatment of psoriasis in Europe for more than 50 years [1]. Most recently, DMF (contained in BG00012/Panaclar) was successfully tested in phase II and III studies of multiple sclerosis (MS) and shown to decrease the frequency of relapses [2]. This promising potential of FAEs in MS prompted to test its efficacy in other degenerative diseases of the central nervous system, in particular in amyotrophic lateral sclerosis (ALS), a lethal motor neuron disorder with currently few therapeutic options.

How FAEs achieve protection in MS remains very uncertain. FAEs exert anti-inflammatory effects through inhibition of pro-inflammatory cytokines [3]. FAEs also exert immunomodulatory effects on dendritic cells [4]. Multiple evidence have shown that FAEs activate the transcription factor nuclear factor (erythroid-derived 2)-related factor 2 (Nrf2) and downstream anti-oxidant pathways including heme-oxigenase 1 (HO-1) and NAD(P)H dehydrogenase quinone 1 (NQO-1) [5–8]. Nrf2 activation is likely due to succination and inactivation of the Nrf2 negative regulator Kelch-like ECH-associated protein 1 (KEAP1) by FAEs [9]. This leads to increased nuclear Nrf2 activity, both *in vivo* and *in vitro* upon FAEs treatment. Importantly, Nrf2 is absolutely required for the protective effects of FAEs during oxidative stress [8,9]. It is thus currently hypothesized that FAEs are protective in MS through their capacity in increasing Nrf2 activity.

FAEs are cell permeant analogs of fumarate, and their application on cultured cells lead to increased intracellular concentrations of fumarate [10]. Interestingly, fumarate has been shown to inhibit the prolyl-hydroxylase domain (PHD) enzymes [11]. PHDs are required for the constitutive degradation of the transcription factor hypoxia-inducible transcription factor 1 alpha (HIF-1α). Upon oxygen deprivation, PHDs inhibition leads to HIF-1α stabilisation and subsequent activation. This in turn activates the expression of a number of target genes required for the adaptation of the cell to low oxygen tension [10,12,13] Upon FAEs application, PHDs inhibition stabilizes HIF-1α leading to activation of its target genes under normoxic conditions [10]. Whether FAEs can activate HIF-1α in the brain in the context of central nervous system (CNS) diseases such as MS is unknown. Interestingly, HIF-1α activation might lead to increased production of VEGF, an angiogenic and neurotrophic factor. VEGF is a highly valuable therapeutic target in amyotrophic lateral sclerosis (ALS). Indeed, mutation of the HIF-1α response element in the VEGF promoter leads to ALS in mouse and VEGF polymorphisms are associated with ALS [14–16]. Moreover, VEGF displays potent protective potential in ALS mouse models [17].

Here we sought to determine whether FAEs are able to activate VEGF in different cell types from the CNS. We show that FAEs induce HIF-1α activation and subsequent VEGF production in astrocytes, while activating Nrf2 in all investigated cell types except microglia. This cell-type specific response to FAEs might be of importance for the protective potential of FAEs.

## Materials and Methods

### Materials

DMEM + GlutaMAX, GlutaMAX, 1xDPBS, penicillin (10.000 Units/mL) and streptomycin (10.000µg/mL) were purchased from GIBCO; poly-L-ornithine hydrobromide, dimethyl sulphoxide Hybri-Max and Trypan Blue Solution were purchased from SIGMA; 1x trypsin-EDTA from PAA; DNaseI from Worthington, Protein Assay from BIORAD; Lipofectamine LTX from Invitrogen; IGF-1-Mouse-ELISA and VEGF-Mouse-ELISA from R&D Systeme; albumin Fraktion V from ROTH; ECL, Super Signal West Pico chemiluminescent substrate for detection HRP from THERMO SCIENTIFIC; Protease Inhibitor Cocktail Tablets “complete Mini EDTA-free” from ROCHE and TMB Substrate Reagent Set was purchased from BIOLOEGEND. Following used plasmids were ordered by ADDGENE; plasmid 27986 (9kB VEGF-luc) [18], plasmid 21103 (PBS/pU6-HIF-1α RNAi plasmid 1) [19], plasmid 21104 (PBS/pU6-HIF-1α RNAi plasmid 2) [19], plasmid 26731 (HRE-luciferase) [20], plasmid 28025 (hrGFP-Keap1) [21]. Mammalian expression vectors, pEF (control vector) and dominant negative Nrf2 (DN Nrf2) were provided by Dr. Jawed Alam (Alton Ochsner Medical Foundation) [22,23].

### Animals

Transgenic male mice bearing the G93A human SOD1 mutation B6.Cg-Tg(SOD1-G93A)1Gur/J were purchased from Jackson Laboratory and bred to female wildtyp mice C57BL/6 purchased from Charles River. Transgenic and nontransgenic offspring were used for further analysis. Genomic DNA was isolated from tail biopsies collected at the 1-5 day-old pups (used for astrocytes-preparation) using the DNeasy genomic DNA isolation kit (Qiagen) following the procedure described by the manufacturer. Genotyping was performed using PCR. SOD and wild type alleles were detected using following primers: SOD 113 (hSOD1-sense) 5’-CAT CAG CCC TAA TCC ATC TGA-3’; SOD 114 (hSOD1-antisense) 5’-CGC GAC TAA CAA TCA AAG TGA-3’; SOD 43 (Interleukin2-sense) 5’-GTA GGT GGA AAT TCT AGC ATC ATC-3’ and SOD 42 (Interleukin2-antisense) 5’-CTA GGC CAC AGA ATT GAA AGA TCT-3’.

All experiments were conducted according to the protocol approved by the Regional Steering Committee Tübingen, Reg. C.0177.

### Cell cultures

To prepare primary astrocytes, neurons, oligodendrocytes and microglia, 1-5 day old transgenic SOD1-G93A mice and their nontransgenic littermates were decapitated. Meninges were removed from the brains, neopallia were dissected and enzymatically (1% Trypsin, Invitrogen, 0,05% DNAse, Worthington, 5 minutes) and mechanically dissociated (oligodendrocytes are digested with papain). The resulting cells were centrifuged (500U/min; 4°C; 10 min), the supernatant discarded, suspended in culture medium (DMEM, 10% FCS [heat-inactivated], 100U/mL penicillin, 100µg/mL streptomycin) and plated into 75-cm^2^ flasks, which were precoated with 1µg/mL poly-ornithine (astrocytes, microglia, neurons) or poly-L-lysine (oligodentrocytes). Cells from one brain were plated into one flask. For getting astrocytes and microglia, adherent cells were washed three times with DPBS and incubated with serum-supplemented culture media after three days. After 7-14 days in culture, microglia cells were manually shaken off, centrifuged (500U/min, 10min), and seeded into 6-well (concentration of 60x10^4^ cells/well) or 96-well plates (concentration of 4x10^4^ cells /well). After 30 minutes, the media were changed to DMEM without phenol red. For neurons, media was changed into Neurobasal medium/B27 after cell plating and after one, four and seven days half of the medium was exchanged and 10 µM cytosine arabinofuranoside were added. For oligodendrocytes the media was exchanged after 3-4h after cell plating. After 3, 6 and 9 days of cell culture 2/3 of the medium were exchanged and 5 µg/ml insulin were added.

For astrocyte cultures, attached cells in the flasks were washed twice with DPBS, detached with 0.05% Trypsin / 0.5mM EDTA, centrifuged (500U/min, 10min) and plated into 6-well (concentration of 10x10^4^ cells/cm^2^) or 96-well plates (concentration of 1x10^4^ cells/cm^2^) in culture media. After 3-5 days when the cells were grown confluently, the media was changed to DMEM without phenol red.

### Treatment of cultures

Using confluent cell monolayers, media were changed into DMEM without phenol red with the same contents, as described. Cells were incubated for 4-24h with the final concentration of 30µM diethyl fumarate (DEF, dissolved in PBS) or dimethyl fumarate (DMF, dissolved in DPBS:DMSO at 1:1) (SIGMA). The HIF-1α-inhibitor YC-1 (final concentration 10µM, dissolved in DMSO), was added 30 minutes before DEF or DMF.

### ELISA for VEGF

The amount of VEGF and IGF-1 was determined with specific ELISAs (R&D Systeme Duo Set) following the manufacturer’s instructions. For ELISA supernatant samples were collected and frozen at -80°C. The remaining cell layers were lysed in 1% Triton/PBS and the total protein amount was quantified by Bio-RAD Dc Protein Assay, following the manufacturer’s instructions. The amount of VEGF and IGF-1 was normalized to the total amount of protein. The concentrations of VEGF and IGF-1 were calculated in pg/mg protein.

### Western Blot

For quantitative Western Blot analysis the medium was removed and the total cell protein extracts were obtained by lysing cells in RIPA-Buffer (50mM Tris, 150mM NaCl, 0.02% NaN_3_, 0.5% NP-40, 0.5% Triton X-100) containing protease inhibitors. Protein content was determined by Protein Assay from Bio-Rad with bovine serum albumin as standard. Cell lysates were electrophoresed on 12% SDS-PAGE under reducing conditions and transferred to a nitrocellulose membrane (Bio-Rad) by standard procedures. Membranes were blocked in PBS containing 3% bovine serum albumin (BSA) for at least 1 hour. After blocking, membranes were incubated with the following primary antibodies: rabbit polyclonal against HIF-1α (Novus Biologicals; 1:500 buffered in 1% BSA; 0,05% NaN_3_ in PBS containing 0,05% Tween 20) or against Nrf2 (Santa Cruz; 1:200 buffered in 1% BSA; 0,05% NaN_3_ in PBS containing 0,05% Tween 20) over night at 4°C. After washing in PBS/0.05% TWEEN 20, membranes were incubated at room temperature for 1h with the secondary antibody (Bio-Rad; 1:5000 in 2.5% non-fat milk powder, goat anti-rabbit IgG-HRP-conjugated) and washed again. Bands were visualized (ECL-immunodetection) using Image Quant LAS4000. Samples were corrected for background and quantified using Image Quant LAS 4000. All values were normalized to housekeeping protein (beta-actin).

### RT-qPCR

After indicated time points, astrocytes, microglia, neurons or oligodendrocytes were harvested and total RNA was extracted using RNA extraction kit (RNeasy Mini Kit, QIAGEN). Complementary cDNA was synthesized from 0.4µg to 1µg of total RNA using the iScript cDNA Synthesis Kit (Bio-Rad). Gene expression of VEGF, Glut1, NQO-1, HO-1, Pol2 and TBP was quantified using the qPCR Mastermix of iQ SYBR Green Supermix^R^ (Bio-Rad). The PCR reactions were performed according the manufacturer’s instructions. The primer sets used for VEGF were (F) 5’-TGA TCA GAC CAT TGA AAC CAC T-3’ and (R) 5’-GGA AGG GTA AGC CAC TCA CA-3’; for Glut1 (F) 5’-ATG GAT CCC AGC AGC AAG-3’ and (R) 5’-CCA GTG TTA TAG CCG AAC TGC-3’; for NQO-1 (F) 5´-AGC GTT CGG TAT TAC GAT CC-3´ and (R) 5´-AGT ACA ATC AGG GCT CTT CTC G-3´; for HO-1 (F) 5´-GTC AAG CAC AGG GTG ACA GA-3´ and (R) 5´-ATC ACC TGC AGC TCC TCA AA-3´ and for the house keeping genes Pol2 (F) 5’-GCT GGG AGA CAT AGC ACC A-3’ and (R) 5’-TTA CTC CCC TGC ATG GTC TC-3’; and for TBP (F) 5’-CGG TCG CGT CAT TTT CTC-3’ and (R) 5’-GGG TTA TCT TCA CAC ACC ATG A-3’. Amplification conditions were set to 3 minutes at 95°C followed by 40 cycles [15 seconds at 95°C, 15 seconds at 60°C] using the real time PCR thermocycler from BioRad (Real Time System CFX 96). All reactions were performed in duplicates. Data were analyzed using the iCycler software and normalized to the normalization factor calculated from the reference genes encoding Pol2 and TBP.

### Transient transfection and luciferase reporter assay

All plasmids were purified by Maxi Prep (EndoFree Plasmid Maxi Kit, Qiagen) using the manufacturer’s instructions.

For transient transfection astrocytes were seeded into 24-well plates with a concentration of 10x10^4^ cells/cm^2^ and grown 24h in cultured media. In brief, for each well to be transfected 3µl lipofectamine LTX and 1µl PLUS REAGENT per 1µg plasmid-DNA was suspended in 100µl DMEM/well. After 10 minutes at room temperature plasmid DNA was added. The mixture was incubated for another 25 minutes at room temperature and then added to the cell culture. Plates were centrifuged for 5 minutes at 500U/min. Cells were incubated for 24h, the transfection complex was removed and then treated with DMF (30µM) or DEF (30µm) for 6h or 18h. After treatment cells were harvested and processed for luciferase activity assay using the luciferase assay system (Promega). Luminescence was measured using a 96-well luminometer (Multilabel Reader PerkinElmer VIKTOR X3).

### Statistical analysis

Statistical analysis was performed using GraphPad version 5.0. Comparison of multiple groups was performed using ANOVA followed by *post-hoc* Newman-Keuls. Significance was considered at p<0.05.

Values are presented as means +/- SEM.

## Results

### FAEs activate HIF-1α target genes in astrocytes

**Figure 1:**
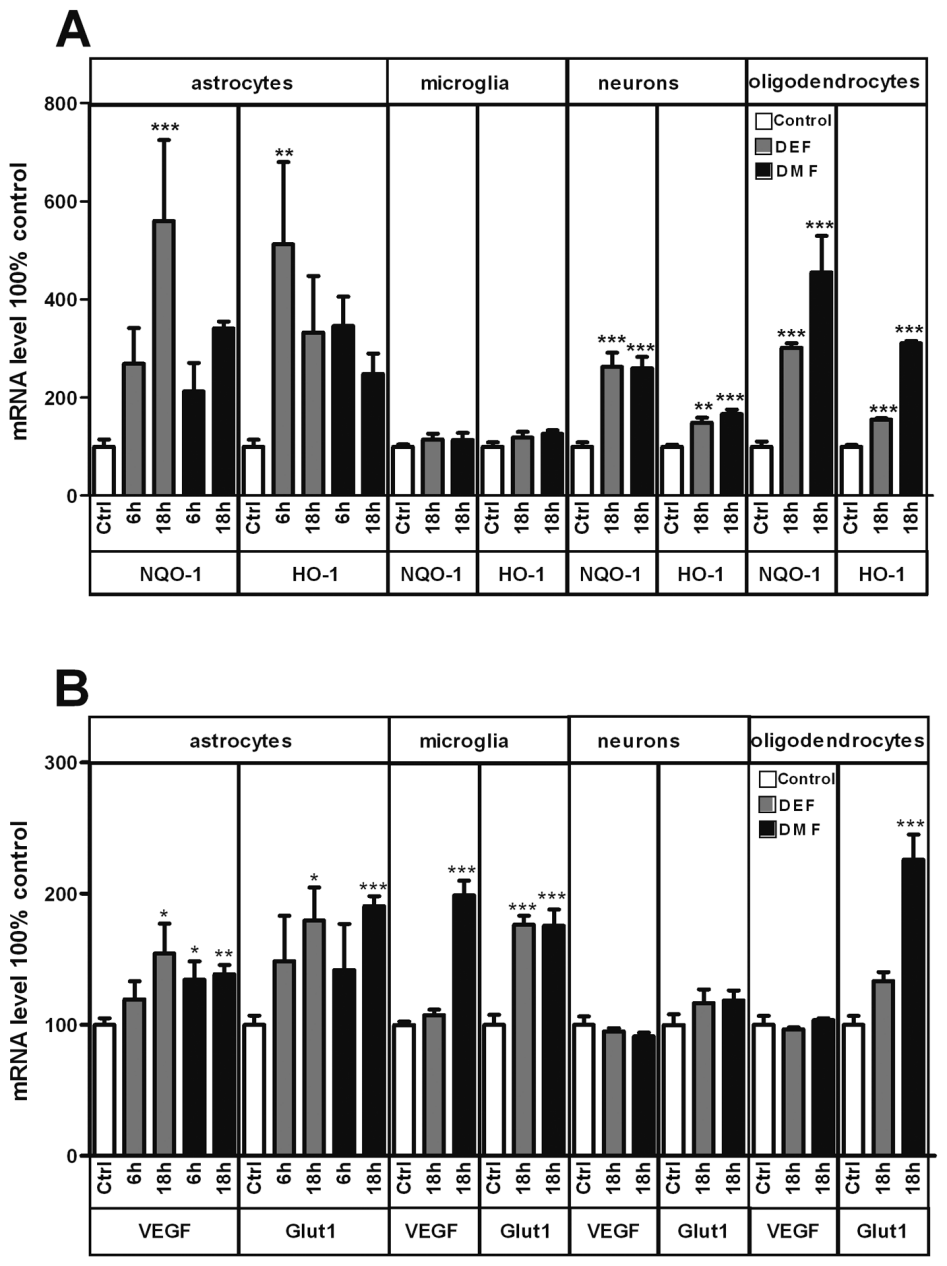
Transcriptional effects of FAEs. (**A**) mRNA levels of NAD(P)H dehydrogenase quinone 1 (NQO-1) and Heme Oxygenase 1 (HO-1), two Nrf2 target genes in astrocytes, microglia, neurons and oligodendrocytes of wild type mice after vehicle (empty columns, Ctrl), or after 6 hours or 18 hours of either 30µM DEF (grey columns, DEF) or DMF (black columns, DMF). Note the robust upregulation of these two genes in all cell types except microglia. (**B**) mRNA levels of vascular endothelial growth factor (VEGF) and glucose transporter 1 (GLUT1), two HIF1-α target genes in astrocytes, microglia, neurons and oligodendrocytes of wild type mice after vehicle (empty columns, Ctrl), or after 6 hours or 18 hours of either 30µM DEF (grey columns, DEF) or DMF (black columns, DMF). FAEs induced the expression of HIF1-α target genes in astrocytes and microglia but not in oligodendrocytes or neurons. *p<0,05; **p<0,01; ***p<0,001; significantly different from corresponding control. Values are mean+/- SEM of n=3 independent experiments.

We hypothesized that FAEs could have differential effects on Nrf2 and HIF-1α pathways in the different CNS cell types. To determine whether this is the case, we screened systematically the expression levels of Nrf2 and HIF-1α target genes in primary cultures of wild type murine astrocytes, microglia, oligodendrocytes and neurons. According to previous studies showing strongly increased Nrf2 activity under these conditions, we stimulated cells for 6 h or 18 h with 30µM of membrane-permeable diethyl- or dimethyl fumarate esters (DEF and DMF) [5,7–9]. Both fumarate esters are converted to fumarate by cellular esterases, and these concentrations are known to double intracellular fumarate levels [10]. Treatment with both FAEs induced robust overexpression of NQO-1 and HO-1, two Nrf2 targets in all cell types except microglia (Figure 1). In contrast FAEs induced the expression of VEGF and GLUT1, two HIF-1α targets in astrocytes and microglia but not in neurons (Figure 1). GLUT1 but not VEGF expression was increased by DMF in oligodendrocytes. Thus, FAEs activate broadly Nrf2 target gene expression, and more cell type specifically HIF-1α target genes.

### FAEs induce HIF-1α accumulation and VEGF release in astrocytes

**Figure 2:**
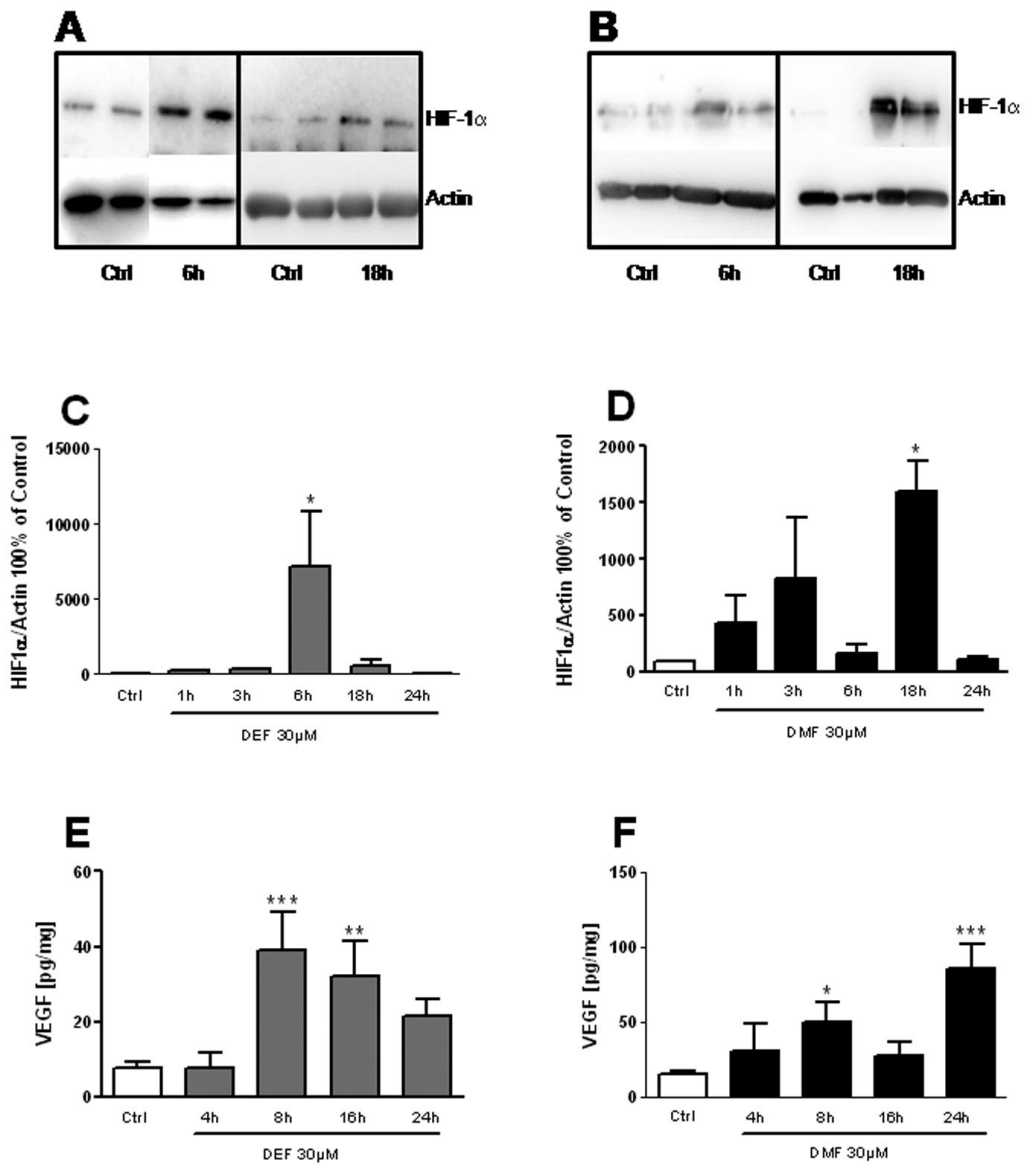
FAEs induce HIF-1α accumulation and VEGF release in astrocytes. Wild type astrocytes were treated with 30µM DEF (A, C, E) or 30µM DMF (B, D, E) for the indicated times. HIF-1α level was measured by Western-Blot (A, B, C, D). VEGF in the supernatant was quantified by ELISA (E, F); *p<0,05; ***p<0,001; significantly different from corresponding control. Values are mean+/-SEM of n=3 independent experiments.

An expected consequence of HIF-1α activation would be release of VEGF. Astrocytes are the major source of VEGF in the CNS and we focused on this cell type. Indeed, microglia did not release detectable VEGF under unstimulated or FAEs stimulated conditions (data not shown) although VEGF mRNA levels were similar, suggesting post-transcriptional regulation of VEGF in this cell type [24,25]. To ascertain that the increase in HIF-1α target mRNA was associated with HIF-1α stabilization, we measured HIF-1α protein levels in response to FAEs in astrocytes. Increasing levels of HIF-1α were observed with DEF (Figure 2A/C) and DMF (Figure 2B/D). Interestingly, the kinetics of HIF-1α accumulation were different between DEF and DMF, with a seemingly biphasic response for DMF and an earlier and stronger response for DEF. This might be due to differences in the metabolism of FAEs. This HIF-1α accumulation was transient and HIF-1α returned to undetectable levels after 24h of treatment (Figure 2C-D). Consistent with previous studies, Nrf2 protein levels were increased upon both DEF or DMF treatments under the same experimental conditions, (Figure S1). HIF-1α accumulation is known to translate into increased mRNA of VEGF and increased VEGF release. Consistently, the application of both FAEs strongly increased the release of VEGF into the culture medium of primary astrocytes (Figure 2E-F). Altogether, our results show that FAEs activate HIF-1α and the HIF-1α associated VEGF release in astrocytes.

### VEGF release upon FAEs is dependent upon HIF-1α and Nrf2 in astrocytes

**Figure 3:**
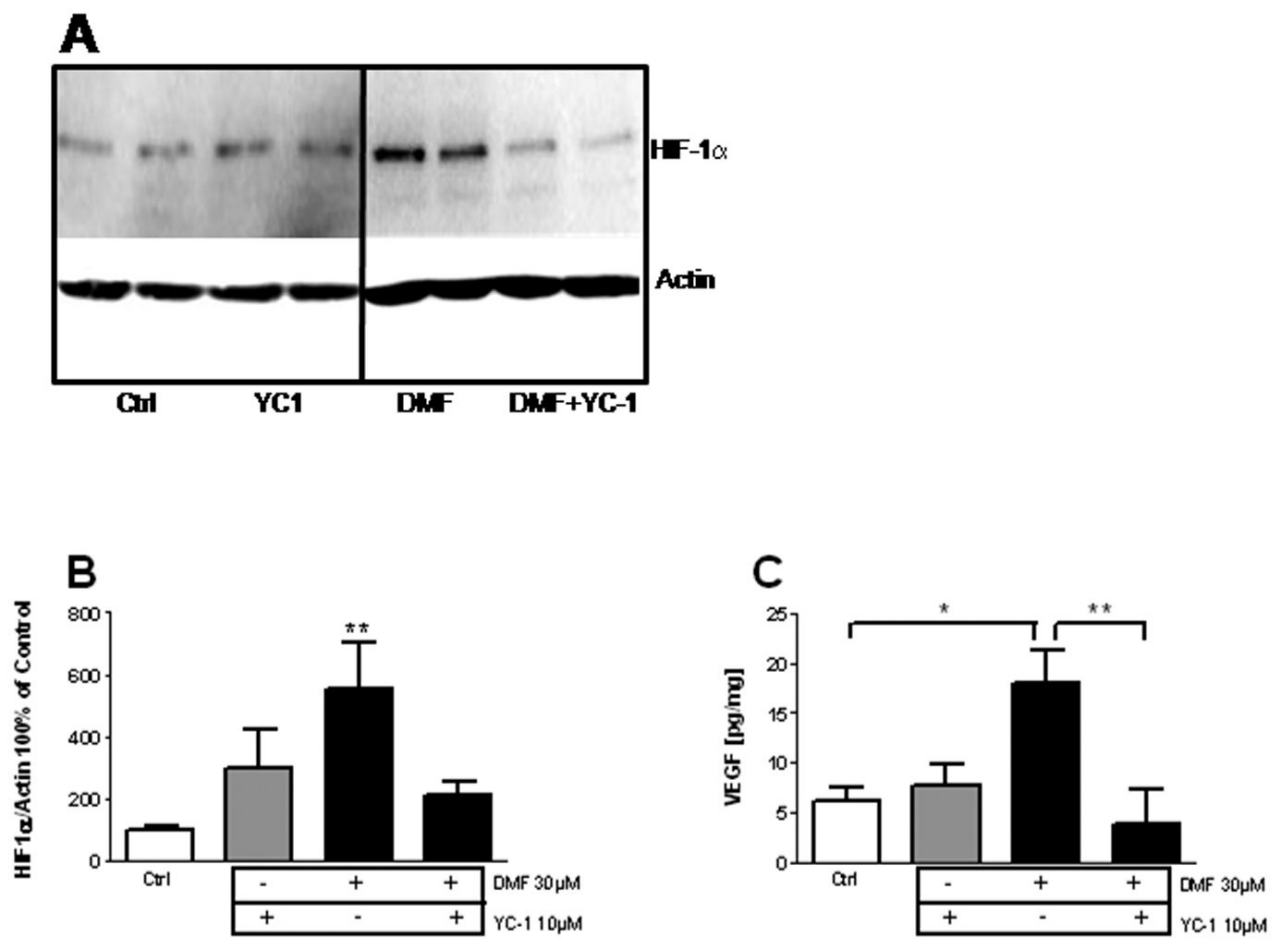
Pharmacological evidence of HIF-1α involvement in VEGF release. Wild type astrocytes were treated with 30µM DMF for indicated times. HIF1-α level was measured by Western-Blot (A) 6h after DMF treatment (B) or 16h after DMF treatment (C). The HIF1-α inhibitor YC-1 was incubated 30min before treatment with DMF. VEGF in the supernatant was quantified by ELISA (C) *p<0,05; ***p<0,001; significantly different from corresponding control. Values are mean+/-SEM of n=3 independent experiments.

**Figure 4:**
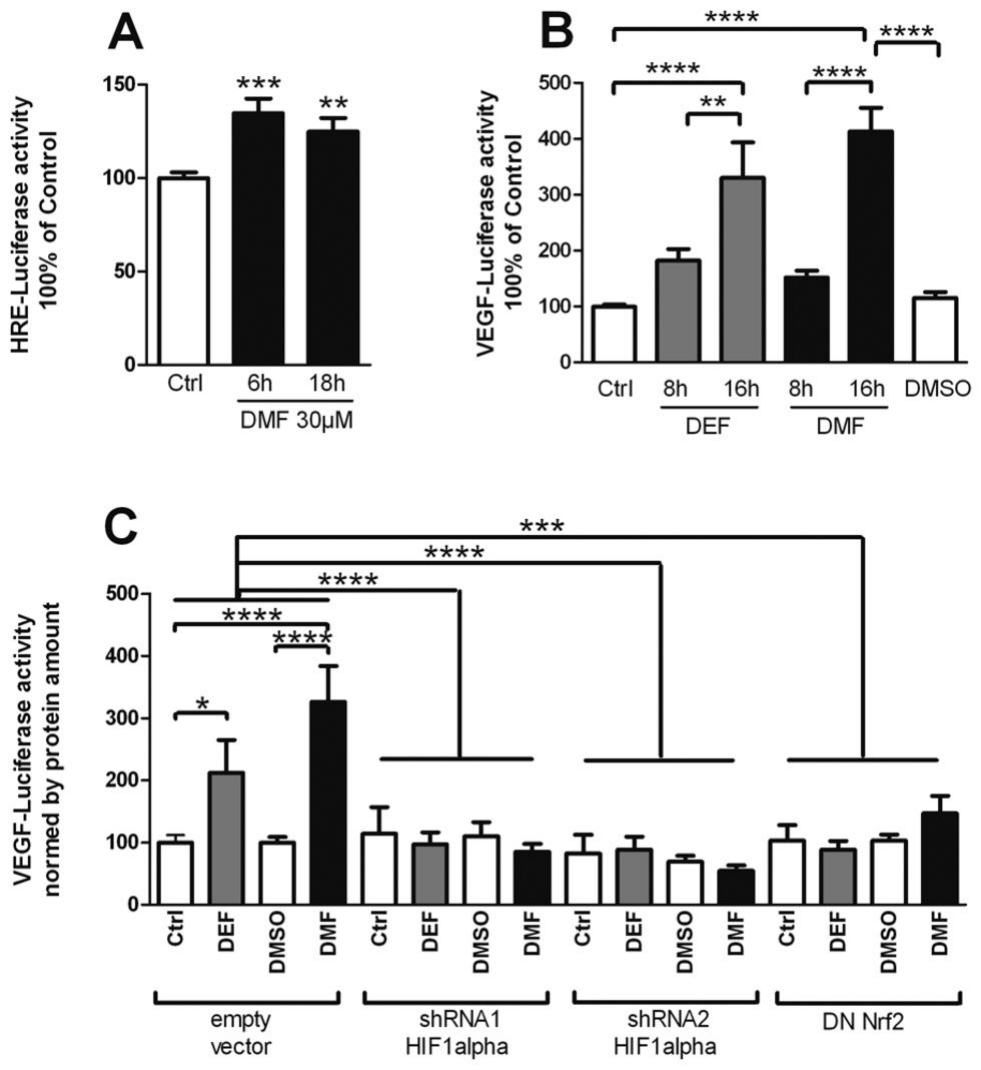
Reporter assay evidence of HIF-1α involvement in VEGF release. Wild type astrocytes were transfected with a HRE-Luciferase reporter plasmid (A), with a VEGF-Luciferase reporter plasmid (B), or were cotransfected with both VEGF-Luciferase plasmid and either an empty vector or a vector encoding a shRNA targeting HIF-1α (two different used, shRNA1 or 2), or an expression vector encoding a dominant negative Nrf2 isoform (C). After 24h cells were treated with 30µM DEF, 30µm DMF or 0,05% DMSO for 6h (A), for 8h (B) or 18h (A-C). Luciferase activity was then measured in cell extract by luminescence. *p<0,05 - ****p<0,0001; significantly different from corresponding control. Values are mean +/- SEM of n=3 independent experiments.

VEGF release and HIF-1α accumulation might be independent events. We pharmacologically inhibited HIF-1α with YC-1 [26–28]. YC-1 inhibits HIF-1α through poorly described mechanisms that include direct destabilization of HIF-1α protein and indirect mechanisms [29]. Pretreatment with YC-1 reverted the accumulation of HIF-1α [30] after DMF treatment (Figure 3A-B), and decreased VEGF release (Figure 3C). YC-1 was unable to decrease HIF-1α accumulation upon DEF treatment (not shown), and this might be due to the earlier and stronger HIF-1α response with DEF as compared with DMF (see Figure 2). To provide further evidence of HIF-1α involvement in FAE-induced VEGF release, we performed reporter assays. FAEs treatment weakly but consistently increased the activity of a luciferase reporter under the control of 6 hypoxia-response elements (HRE) selectively activated by HIF-1α (Figure 4A). When luciferase was placed under the control of a 9kb VEGF promoter, the induction by FAEs was more potent (Figure 4B), suggesting the involvement of other transcription factors than HIF-1α. shRNA knock-down of HIF-1α reverted VEGF promoter activity showing that HIF-1α is necessary for VEGF induction in response to FAEs (Figure 4C). Since FAEs also increase Nrf2 activity in astrocytes, we probed for Nrf2 involvement. Expression of a dominant-negative isoform of Nrf2 also decreased the VEGF promoter activity elicited by FAEs (Figure 4C). Thus, HIF-1α and Nrf2 cooperate to activate VEGF release in astrocytes upon FAEs treatment.

### ALS astrocytes display a reduced release in VEGF in response to FAEs

**Figure 5:**
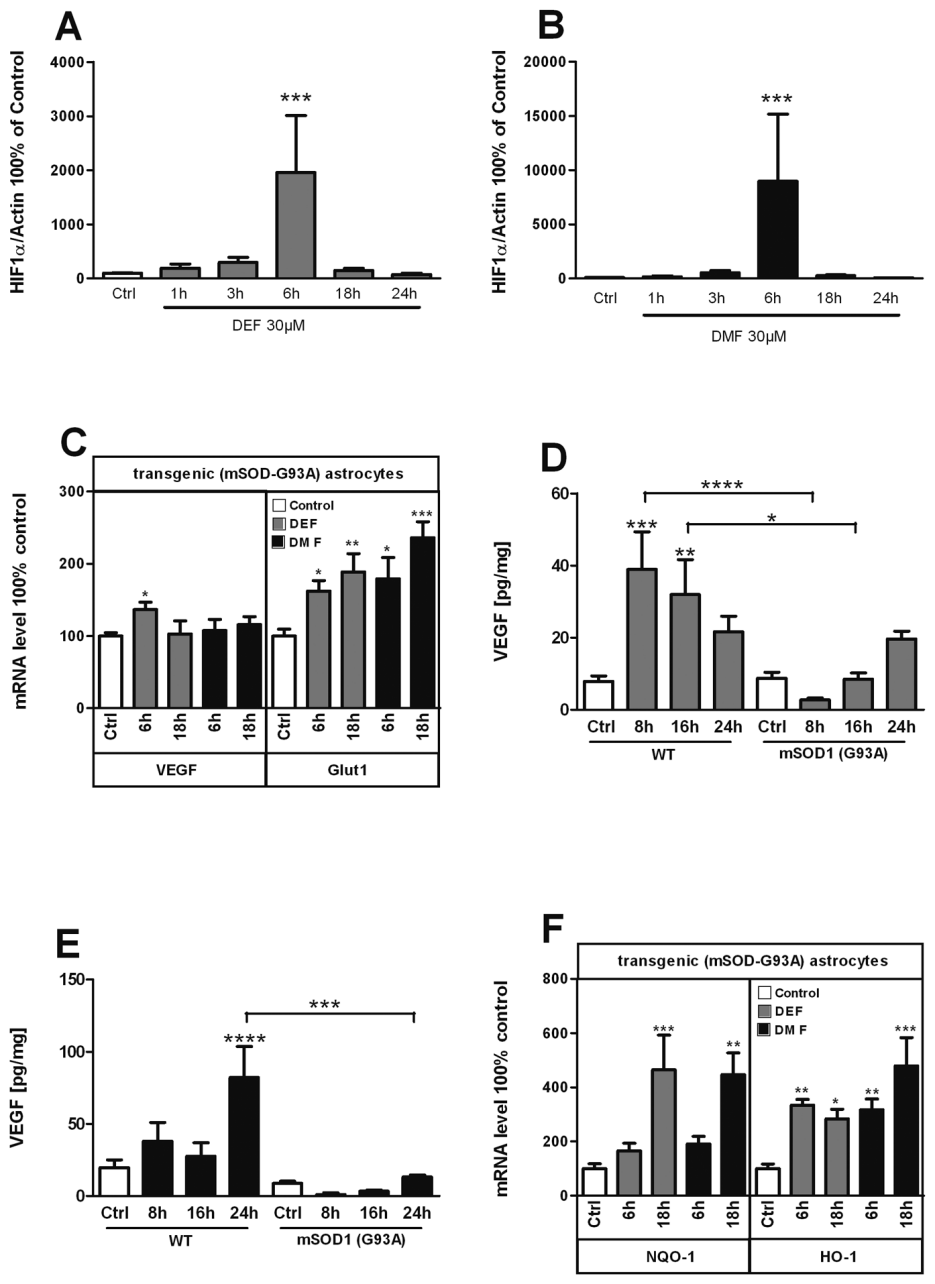
Transgenic (mSOD-G93A) astrocytes show abnormal response to FAEs. Transgenic (mSOD-G93A) astrocytes were treated with 30µM DEF (A,C,D,F) or 30µM DMF (B,C,E,F) for indicated times and the following read-outs were measured; HIF-1α level by Western-Blot (A,B); HIF-1α (VEGF, GLUT1) target genes using qPCR (C). VEGF release as determined by ELISA (D,E); and Nrf2 target genes (NQO-1, HO-1) (F) *p<0,05; **p<0,01; ***p<0,001; significantly different from corresponding control. Values are mean +/- SEM of n=3 independent experiments.

At this point of our studies, we had shown that FAEs display a novel pharmacological action on astrocytes leading to increased VEGF release. Increasing VEGF release by astrocytes is of direct therapeutic relevance for ALS. Indeed, impaired hypoxic response is a feature of ALS [31] and VEGF delivery is strongly neuroprotective in animal models [14,17]. To provide evidence that FAEs could be of therapeutic interest in ALS, we first sought to determine whether these drugs were able to increase VEGF release in astrocytes derived from an animal model of ALS. To this aim, we cultured astrocytes from transgenic SOD1(G93A) mice, a well documented animal model of ALS to assay for FAEs response. While wild type astrocytes strongly accumulate HIF-1α when stimulated with either DEF or DMF, SOD1(G93A) astrocytes had a blunted and delayed response to both FAEs (Figure 5A-B). Consistently, the VEGF mRNA expression (Figure 5C) and VEGF release (Figure 5D) upon FAE stimulation were much weaker in transgenic astrocytes than in wild type astrocytes. Interestingly, while HIF-1α activation by FAEs was blunted, Nrf2 dependent gene expression (Figure 5F) and protein levels (Figure S2) were similar in wild type and SOD1(G93A) astrocytes. Thus, astrocytes from a transgenic model of ALS are less responsive to FAE-induced HIF-1α activation (Figure 5C-D) but retain the activation of Nrf2.

## Discussion

Here, we show that FAEs are able to differentially activate HIF-1α and Nrf2 in cell types of the CNS. We further show that HIF-1α activation elicited by FAEs lead to release of VEGF by astrocytes and that this is likely to be potentiated by Nrf2. Last, we observed that astrocytes from a transgenic mouse model of ALS are much less sensitive to FAEs induced HIF-1α activation.

### FAEs show differential effects among cell types

Previous work indicated that FAEs were able to activate Nrf2 in neurons and astrocytes [5,7–9,32,33] and that this Nrf2 activation is at least partially responsible for the cytoprotective potential of these compounds [8,9]. However, whether this Nrf2 activation also occurs in microglia or oligodendrocytes, which are important players in MS and ALS was unknown. Here we show that FAEs activate Nrf2 in most CNS cell types, excluding microglia. Apart from Nrf2 activation, FAEs have been shown to activate HIF-1α through inhibition of prolyl hydroxylases in cell lines [10]. This likely occurs through the release of fumarate by hydrolysis of FAEs, although the intracellular metabolism of FAEs is poorly documented [10]. Here we show that this HIF-1α activation occurs in primary cells but only in some cell types (astrocytes and microglia). Neurons did not activate HIF-1α upon FAE exposure while oligodendrocytes displayed a late and partial response. The reason for this difference in cell sensitivity is unknown, but astrocytes and microglia are known to be sensitive to hypoxia. Of note astrocytes display a strong metabolic flexibility as compared with neurons. In particular, astrocytes are able to strongly activate glycolysis upon mitochondrial inhibition, which is not the case for neurons [34]. This intrinsic metabolic difference might underlie the differential sensitivity of astrocytes to FAEs as compared to neurons. Further research is needed to elucidate this point.

### FAEs activate HIF-1α leading to astrocytic VEGF release

In our study, we show that FAEs induce HIF-1α activation in astrocytes and subsequent VEGF production. FAEs are able to stabilize the HIF-1α subunit of the HIF complex, and subsequently increase the expression of VEGF and GLUT target genes. This was associated with increased VEGF release in astrocytes. In contrast to astrocytes in microglia FAEs induced not a release of VEGF, even though HIF-1α was activated and VEGF mRNA was produced. This might be associated with a post-transcriptional regulation specific for microglia [24,25,35]

Our pharmacological and shRNA experiments converge to demonstrate that HIF-1α is required for the transcriptional activation of VEGF by FAEs, and presumably of increased VEGF release in astrocytes. Fumarate, an intermediate of the Tri-carboxylic acid cycle, activates HIF-1α through inhibition of PHDs [11]. It is thus likely that the same mechanism accounts for astrocytic HIF-1α accumulation in response to FAEs since FAEs application doubles fumarate intracellular levels [10]. While HIF-1α is required for VEGF transcriptional activation, its sole activation is not sufficient to account for the full blown effect. Indeed, the FAEs-increased reporter activity detected with 6 x HRE, that is only activated through HIF-1α, is much weaker than when using the full VEGF promoter. This suggests that other transcription factors are involved in the observed effect. Our data further indicate that one of these additional transcription factors is Nrf2, since the overexpression of a dominant-negative Nrf2 abolishes the FAE-mediated activation of VEGF. The effect of Nrf2 on VEGF transcription remains unknown, in particular, whether it is direct or indirect. Other transcription factors or transcriptional co-activators might be involved, in particular PGC-1α, that mediates VEGF activation in ischemic muscle independently of HIF-1α [36]. Our study illustrates a potential beneficial effect of HIF-1α activation on neuronal survival. However, the role of astrocytic HIF-1α is more complex. In particular astrocytic HIF-1α has been shown to be deleterious for neuronal survival in cellular models of hypoxia [37] and VEGF release due to HIF-1α activation is deleterious in vivo for animal models of EAE [38]

### ALS astrocytes show blunted sensitivity to FAEs

A third major result of our study is that astrocytes from SOD1(G93A) mice exhibit strongly reduced HIF-1α accumulation in response to FAEs. A large body of litterature has previously linked ALS and HIF-1α. First, a deletion of HRE in the *Vegf* murine gene leads to reduced VEGF levels and ALS-like disease [15]. Conversely, increasing VEGF through either gene therapy [17] or intracerebrovascular delivery [39] increases the survival of SOD1(G93A) mice. Thus, a straightforward therapeutic strategy would be to pharmacologically increase VEGF expression, in particular in astrocytes that produce most of brain VEGF. In this respect, FAEs would be strong candidates for such a therapeutic strategy since they are able to promote VEGF expression and release by wild type astrocytes. However, while FAEs are able to induce HIF-1α stabilization in SOD1(G93A) astrocytes, they fail to increase VEGF mRNA levels or VEGF release after stimulation with FAEs. Indeed, in basal conditions, transgenic astrocytes release less VEGF than wild type astrocytes. These data suggest that mutant SOD1 expression impairs the signalling from HIF-1α to transcriptional activiation. These data mirror that obtained in peripheral monocytes of ALS patients that display a blunted hypoxic response [16] and are consistent with the observation that ALS patients display paradoxical regulation of VEGF in hypoxia [40]. It is likely that ALS-related events, in particular expression of mutant SOD1, deregulates HIF-1α signalling, downstream of HIF-1α stabilization, through yet unknown mechanisms. These results cast doubts on the utility as a therapeutic strategy of drugs stimulating VEGF release through HIF-1α stabilization in ALS.

Altogether, we show here that FAEs are able to elicit differential transduction pathways depending on cell types, that include Nrf2 and HIF-1α, and converge, at least in astrocytes, to promote a pseudo-hypoxic like response with increased VEGF expression and release. ALS mutant cells are unable to respond properly to FAEs, suggesting that disease intrinsic mechanisms are involved in FAE response.

## Supporting Information

Figure S1 **FAEs induce Nrf2 in astrocytes.** Wild type astrocytes were treated with 30µM DEF (A, C) or 30µM DMF (B, D) for the indicated times. Nrf2 levels were measured in duplicates by western blot. *, p<0.05, **, p<0.01 significantly different from corresponding control (ANOVA followed by post-hoc Newman-Keuls). Values are the means +/- SEM of n=3 independent experiments.
(TIFF)

Figure S2 **FAEs induce Nrf2 in transgenic ALS astrocytes.** Transgenic (mSOD1-G93A) astrocytes were treated with 30µM DEF (A, C) or 30µM DMF (B, D) for the indicated times. Nrf2 levels were measured in duplicates by western blot. *, p<0.05, **, p<0.01 significantly different from corresponding control (ANOVA followed by *post-hoc* Newman-Keuls). Values are the means +/- SEM of n=2 independent experiments.
(TIFF)
