# Supplementary figures and images for: Fumaric Acid Esters Stimulate Astrocytic VEGF Expression through HIF-1α and Nrf2
Source: PLoS One. 2013 Oct 3;8(10):e76670. doi: 10.1371/journal.pone.0076670 (PMC3789659; doi:10.1371/journal.pone.0076670)

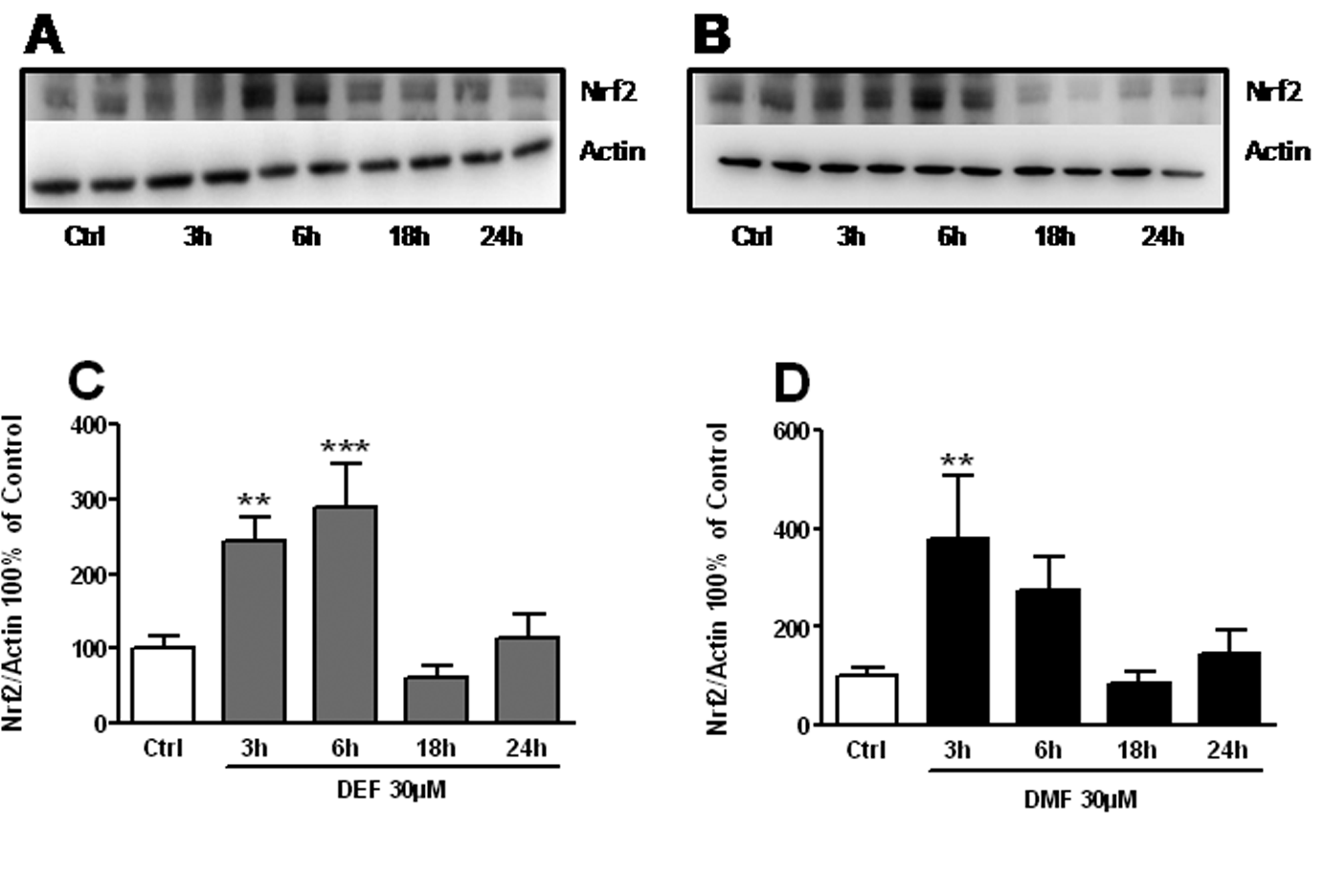

Supplement: Figure S1 — FAEs induce Nrf2 in astrocytes. Wild type astrocytes were treated with 30µM DEF (A, C) or 30µM DMF (B, D) for the indicated times. Nrf2 levels were measured in duplicates by western blot. *, p<0.05, **, p<0.01 significantly different from corresponding control (ANOVA followed by post-hoc Newman-Keuls). Values are the means +/- SEM of n=3 independent experiments. (TIFF) [file pone.0076670.s001.tiff]

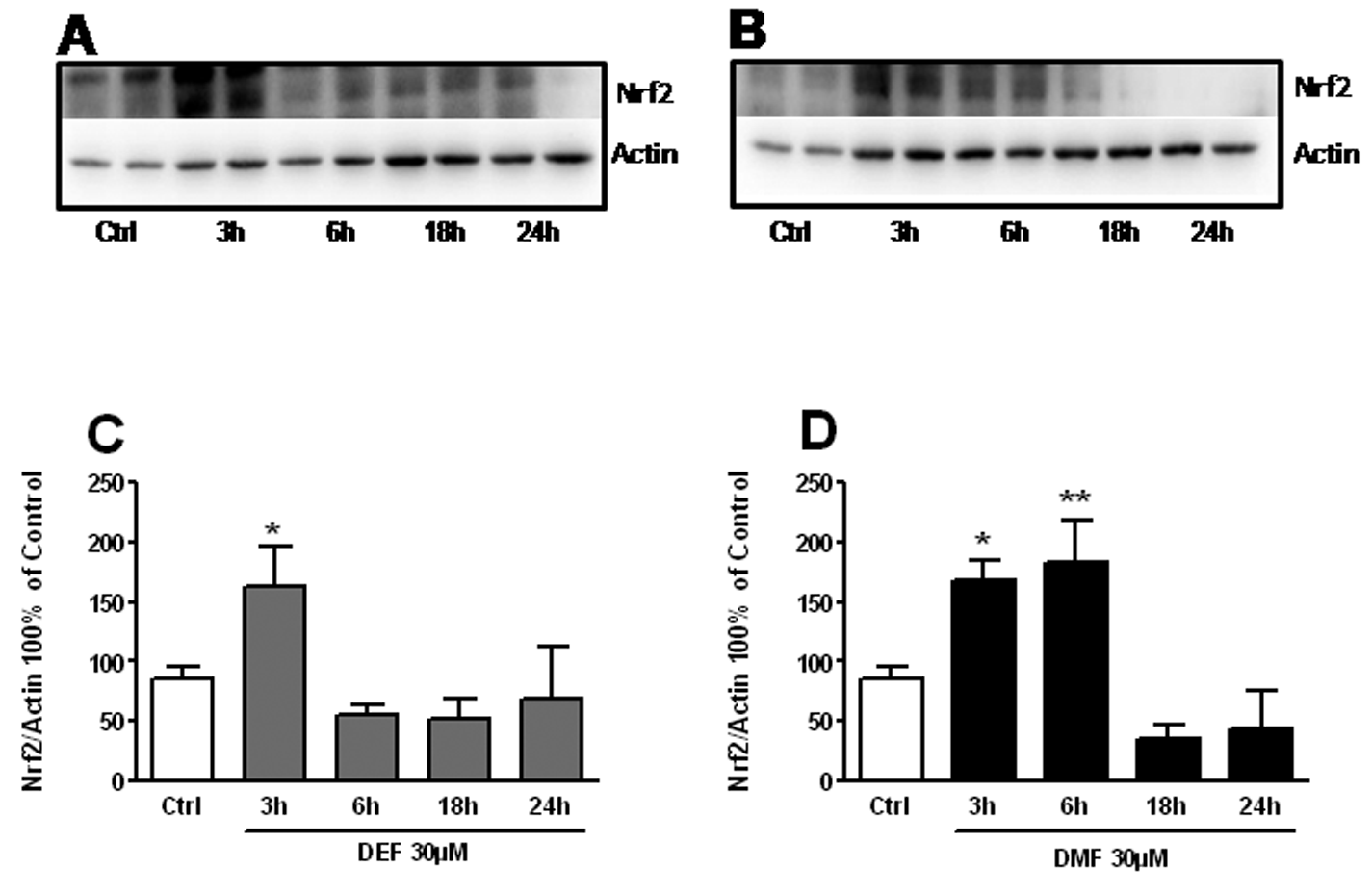

Supplement: Figure S2 — FAEs induce Nrf2 in transgenic ALS astrocytes. Transgenic (mSOD1-G93A) astrocytes were treated with 30µM DEF (A, C) or 30µM DMF (B, D) for the indicated times. Nrf2 levels were measured in duplicates by western blot. *, p<0.05, **, p<0.01 significantly different from corresponding control (ANOVA followed by post-hoc Newman-Keuls). Values are the means +/- SEM of n=2 independent experiments. (TIFF) [file pone.0076670.s002.tiff]
